# Parity and Season Affect the Performance of an Automated Activity Monitoring System for Estrus Detection in Grazing Girolando Cows

**DOI:** 10.1155/vmi/8201912

**Published:** 2026-05-29

**Authors:** Sara Adna Santos De Oliveira, Ricardo Alamino Figueiredo, João Henrique Moreira Viana, Livia Loiola Dos Santos Féres, Luiz Fernando Rodrigues Féres, Ricarda Maria Dos Santos, Carlos Frederico Martins, Isabel Cristina Ferreira

**Affiliations:** ^1^ Campus Gloria, Federal University of Uberlandia, BR-050 KM 78, 38410-337, Uberlandia, Minas Gerais, Brazil, ufu.br; ^2^ Embrapa Genetic Resources and Biotechnology, Brazilian Agricultural Research Corporation, Federal, Brasilia, 70770-900, Brazil, embrapa.br; ^3^ EPAMIG (Agricultural Research Agency of the State of Minas Gerais), Rua Afonso Rato 1301 - Bairro Mercês. CEP, 38.001-970, Uberaba, Minas Gerais, Brazil; ^4^ Brazilian Agricultural Research Corporation, Embrapa Cerrados, Federal, Brasilia, 73310-910, Brazil, embrapa.br

**Keywords:** activity sensors, animal reproduction, Zebu dairy cows

## Abstract

Accurate estrus detection in grazing crossbred dairy cows (Zebu × Taurine) remains challenging in Brazil, and automated activity monitoring (AAM) systems may be a viable alternative. This study evaluated the effects of management and environmental factors on AAM performance for estrus detection in grazing Girolando cows. Nonpregnant 5/8 Gir × Holstein cows (*n* = 25) were monitored during both dry and rainy seasons. During the dry season, cows were managed in semiconfinement with corn silage supplementation. In the rainy season, they grazed *Panicum maximum* cv. Mombaça pastures with or without eucalyptus trees. Each cow wore an AAM collar (CowMed, Brazil), and after individual baseline calibration, cows followed a timed artificial insemination (TAI) protocol. A statistical model included season, location, parity, and detection method (AAM, visual observation [VO], adhesive patches [APs]) as fixed effects. Cow and cow × season interaction were random effects. Multiparous cows had higher estrus detection probabilities (97%) compared to primiparous cows (85%), regardless of method, location, or season. AAM detection probability was higher during the rainy season (98%) than during the dry season (73%). For AP, the detection probability was 80% in the rainy season and 97% in the dry season. VO detection remained stable at 92% in the dry season and 95% in the rainy season. Based on corpus luteum (CL) presence, AAM sensitivity and accuracy were 85% and 75%, respectively, while specificity was 33%. Parity and seasonal management significantly influenced AAM performance. The generally low specificity across detection methods highlights the need for improved algorithms and integrated estrus detection strategies in grazing systems.

## 1. Introduction

Reproductive efficiency is a key determinant of dairy farm profitability. Management failures extend calving intervals, decrease milk yield, and increase replacement costs. Estrus remains a key bottleneck affecting reproductive performance in dairy herds. Conventional estrus detection methods, such as visual observation (VO), are conceptually simple. However, they require skilled labor, are time‐consuming, and are heavily influenced by factors like lameness, herd size, nutrition, and facility design [1]. Additionally, VO depends on the observer and is generally limited to daylight hours [2]. Auxiliary methods, such as heat detection patches (HDP; adhesive tapes attached to the tail base), can assist in identifying cows in estrus [3]. However, if the device is used incorrectly or lost, its reliability is reduced, which can prevent confirmation of mounting activity.

Hormonal protocols for fixed time artificial insemination (FTAI) provide an alternative to traditional estrus detection. These protocols synchronize ovulation, eliminating the need for direct estrus detection, and are widely adopted in dairy and beef systems [4]. Nonetheless, consumer demand for hormone‐free products has increased interest in nonpharmacological reproductive strategies [5].

In recent decades, automated activity monitoring (AAM) sensors have rapidly expanded in dairy production, enabling continuous monitoring of cow behavior and offering valuable insights into estrus, health, nutrition, and animal welfare [6]. By detecting individual behavioral changes, AAM sensors can overcome many limitations of VO. Although high AAM sensitivity (ranging from 89% to 97%) has been mainly reported in confined systems [7, 8], system performance under grazing conditions remains inconsistent and warrants further research [9, 10].

It is unknown whether the presence of trees in pasture areas may restrict signal transmission or change the expression of estrus, or whether environmental conditions across seasons, combined with their respective feeding management, might influence cow behavior and, consequently, the effectiveness of estrus detection by AAM sensors. This study aimed to determine the effects of management (seasonal feeding) and environmental factors (location and parity) on the performance of an AAM system compared with conventional estrus detection methods in grazing Girolando cows. We hypothesized that the AAM system could achieve performance, in terms of sensitivity and specificity, equivalent to that of conventional methods, even under the environmental and management variability inherent to extensive systems, thereby increasing reproductive efficiency in pasture‐based production systems limited by labor shortages.

## 2. Materials and Methods

### 2.1. Study Location and Animals

This longitudinal study was conducted at Embrapa Cerrados (Center of Technology for Dairy Zebu Breeds—CTZL, 15°57′09″ S, 48°08′12″ W), located in Brasília, Federal District, Brazil. Lactating, nonpregnant 5/8 Holstein  × Gir (*Bos taurus taurus* × *Bos taurus indicus*) cows were used (*n* = 25), including 13 primiparous and 12 multiparous animals. The cows had an average body weight of 500 ± 10 kg, 205 ± 129 days in milk (DIM), and an average milk yield of 14.9 kg/day.

Before enrollment, all cows underwent reproductive evaluation using transrectal ultrasonography (HS 1500 equipped with a 7.5‐MHz linear transducer [Honda, Japan]), and only animals without uterine and ovarian abnormalities were included in the experiment.

### 2.2. Experimental Design and Environmental Conditions

Estrus evaluations were conducted during two experimental periods: September 2021 (dry season; temperature–humidity index [THI] = 71 ± 4) and November 2021 (rainy season, THI = 70 ± 4). In both seasons, cows were evenly allocated to one of two pasture environments: (1) a silvopastoral system, consisting of pasture intercropped with eucalyptus trees approximately 30 m tall, arranged in single rows spaced 25 m apart; (2) or open pasture without trees (full sun).

### 2.3. Reproductive and Feeding Management

During the dry season, cows were managed under a semiconfinement system on pasture and received ad libitum corn silage‐based diets containing 8% crude protein (CP) and 65% total digestible nutrients (TDNs). Silage was delivered in uncovered central feed bunks within 900‐m^2^ paddocks, with free access to water and mineral supplements. Silage supply followed NRC recommendations, with amounts adjusted to maintain approximately 10% refusals. Although dry pasture was available, corn silage remained the primary forage source.

During the rainy season, cows grazed on *Panicum maximum* cv Mombaça pastures (12.8% CP and 65% TDN) under a rotational grazing system, with a stocking rate of 13 animals per paddock (0.66 ha each), covering a total of 16 ha (8 ha under silvopastoral conditions and 8 ha in open pasture). Mineral salt was freely available.

In both seasons, a corn‐ and soybean‐based concentrate (22% CP and 80% TDN) was given individually during milking at a ratio of 1 kg of concentrate per 3 kg of milk produced.

Cows were milked twice daily in the presence of their calves, at a pit‐parlor equipped with a mechanical milking system. After milking, the cows returned to pasture paddocks located approximately 900 m from the milking parlor. All experimental procedures were approved by the Ethics Committee in Animal Use of Embrapa (CEUA protocol no. 533.2541‐1/2017).

### 2.4. Estrus Detection Methods

Estrus detection was evaluated on Days 9, 10, and 11 of the timed artificial insemination (TAI) protocol using three methods: (1) AAM, (2) VOs, and (3) adhesive patches (APs). Daily estrus detection outcomes across all methods were compared.

Estrus occurrence was confirmed by the presence of a corpus luteum (CL) detected via transrectal ultrasonography 8 days after the protocol (D 18), which was adopted as the physiological reference standard [11, 12]. This approach allows confirmation of ovulation independently of behavioral expression.

The same 25 cows were assessed in both seasons, resulting in repeated observations over time. However, 14 evaluations conducted during the rainy season were excluded due to poor image quality, which prevented reliable identification of CL. As a result, the performance analysis was based on 36 confirmed observations: 25 in the dry season and 11 in the rainy season.

## 3. FTAI Protocol

The FTAI protocol involved an intramuscular (i.m.) injection of 2 mg of estradiol benzoate (Agener União Saúde Animal, Embu‐Guaçu, SP, Brazil) and insertion of a 1‐g progesterone intravaginal device (Ourofino Saúde Animal, São Paulo, SP, Brazil) on Day 0 (D0). On Day 8 (D8), the device was removed, and cows received i.m. injections of 0.5 mg sodium cloprostenol (JA Saúde Animal, Patrocínio Paulista, SP, Brazil), 400 IU of equine chorionic gonadotropin (eCG; Ecegon; Biogénesis Bagó Saúde Animal, Curitiba, PR, Brazil), and 1 g of estradiol cypionate (Ourofino Saúde Animal, Cravinhos, SP, Brazil). APs were applied to the base of the tail immediately after device removal.

### 3.1. AAM

For AAM‐based estrus detection, two antennas were installed: (1) a primary C‐Com v2.0 IMOB antenna at the entrance of the milking parlor to capture individual baseline patterns and (2) a secondary C‐Com v1 IMOB (C‐COM1) antenna positioned approximately 400 m from the center of the paddock. Cows were fitted with AAM neck collars (CowMed, Brazil) 15 days before the FTAI protocol to establish individual behavioral baselines.

The collars had a waterproof accelerometer mounted on the upper left side of the neck, with a counterweight to ensure optimal sensor orientation. The system continuously recorded locomotor activity, rumination, and idleness. Estrus alerts were generated by the algorithm when increased locomotor activity coincided with decreased rumination compared to individual baseline patterns.

### 3.2. VO

Visual estrus detection was performed by a single trained observer on Days 9, 10, and 11 of the FTAI protocol, twice daily (at 08:00 and 16:00), for 1 h per observation period. Primary estrus signs (standing to be mounted) and secondary signs (clear vaginal discharge, agitation, raised tail, and increased social interactions) were recorded.

### 3.3. APs

Commercial APs (EstrotecTM, USA) were attached transversely to the dorsal base of the tail for estrus detection based on color change. Before application on D8, the patches were preheated in the compartment to activate the adhesive. The tail head area was brushed and cleaned to remove loose hair, moisture, and grease. Estrus was considered detected when at least 75% of the silver coating had been removed due to mounting activity.

### 3.4. Statistical Analysis

The statistical analysis was conducted using the PROC GLIMMIX procedure in SAS Studio (SAS Institute, Inc., Cary, NC, USA). A generalized mixed model with a binomial distribution and logit link function was employed to estimate the probability of estrus detection. Fixed effects included season (dry vs. rainy), location (silvopastoral vs. open pasture), parity (primiparous vs. multiparous), and detection method (AAM, VO, and AP). To account for repeated measurements within animals, the cow was included as a random effect, along with the cow × season interaction to model individual variability across seasons.

An initial full model, including all possible interactions among the fixed factors, was tested. Due to convergence limitations, interactions were subsequently evaluated individually while retaining all main effects. Only interactions that were statistically significant in addition to main effects were included in the final analyses. *p* values for all tested models are provided in the Supporting Material (Table S1).

Adjusted probabilities of estrus detection were derived by transforming the LS‐means from the GLIMMIX model using the inverse logit function (*p* = *e* ^*n*^/1 + *e* ^*n*^).

The overall performance of the AAM system during the rainy season was evaluated by calculating the following parameters: sensitivity, accuracy, and specificity. Sensitivity was calculated as the proportion of cows with a detected CL that generated an AAM alert. Accuracy was defined as the proportion of cows correctly classified as either in estrus (CL present with alert) or not in estrus (CL absent without alert). Specificity was defined as the proportion of cows confirmed not to be in estrus (absence of CL) that did not generate an AAM alert.

## 4. Results

### 4.1. Fixed Effects and Interactions

Parity significantly affected estrus detection probability (*F* = 4.38; *p* = 0.0391), indicating differences between primiparous and multiparous cows. The most notable result was a significant interaction between season and detection method (*F* = 4.71; *p* = 0.0112), indicating that detection efficiency varied markedly across environmental conditions. No significant main effects were observed for detection method (*F* = 0.07; *p* = 0.9279), season (*F* = 0.34; *p* = 0.5646), or location (*F* = 1.92; *p* = 0.1690) (Table 1). These findings indicate that while the detection method alone did not determine estrus detection success, its performance was strongly modulated by seasonal conditions, and parity played a critical role in estrus expression and detection.

**TABLE 1:** Fixed effects and *F* statistics on estrus occurrence in 25 Girolando cows.

Fixed effects	*df*	*F* statistics	*p* values
Method	2	0.07	0.9279
Season	1	0.34	0.5646
Location	1	1.92	0.1690
Parity	1	4.38	0.0391
Season × method	2	4.71	0.0112

### 4.2. Odds Ratio (OR) Analysis (Parity Effect)

OR analysis further confirmed the influence of parity on estrus detection. Primiparous cows had an OR of 0.171 compared to multiparous cows (95% confidence interval not including 1.0, Table 2), indicating an 82.9% lower likelihood of detecting estrus, independent of other model factors. In contrast, OR estimates for season, location, and detection method were not statistically significant, as their confidence intervals included 1.0.

**TABLE 2:** Odds ratios and 95% confidence intervals for the effects of season, parity, location, and detection method (adhesive patches [AP], automated activity monitoring automatic [AAM], and visual observation [VO]) on estrus occurrence in 25 grazing Girolando cows.

Fixed effects	Categories	Odds ratio (estimate)	Confidence limit 95% lower	Confidence limit 95% upper
Season	Rainy vs. dry	0.624	0.123	3.168

Parity	Multiparous vs. primiparous	0.171	0.032	0.914

Location	Sun × shade	3.034	0.619	14.877

Method	VO × AP	0.786	0.158	3.911
	VO × AAM	0.754	0.151	3.755
	AAM × AP	1.043	0.185	5.872

### 4.3. Adjusted Probabilities and Sensitivity

Adjusted probabilities of estrus detection and diagnostic performance metrics are shown in Table 3. These results confirm the significant interaction between detection method and season. Overall, high adjusted probabilities were observed across most methods and seasons, especially for AAM and VO during the rainy season and for AP during the dry season.

**TABLE 3:** Adjusted probabilities (and 95% confidence intervals) for estrus detection in 25 grazing Girolando cows, based on detection methods (AP, AAM, and VO), season, location, and parity.

Method^∗^	Season	Adjusted probabilities means	Confidence interval (95%)
AP	Dry	0.97	(0.80–0.99)
AP	Rainy	0.78	(0.52–0.92)
AAM	Dry	0.70	(0.45–0.87)
AAM	Rainy	0.98	(0.83–0.99)
VO	Dry	0.91	(0.71–0.98)
VO	Rainy	0.95	(0.77–0.99)
Parity			
Primiparous		0.85	(0.69–0.93)
Multiparous		0.97	(0.88–0.99)
Local			
Sun		0.96	(0.86–0.98)
Shade		0.88	(0.74–0.95)

^∗^Adhesive patches (AP), automated activity monitoring (AAM), visual observation (VO).

Model‐predicted estrus detection probabilities for the AAM system were 98% in the rainy season and 70% in the dry season. For the AP method, expected probabilities were 78% in the rainy season and 97% in the dry season. In contrast, VO showed consistently high and stable detection probabilities across seasons (91% in the dry season and 95% in the rainy season) (Table 3). It is important to note that these estimates reflect the experimental conditions, considering the effects of parity, location, and the random effects of individual cows. Sensitivity values above 90% are generally regarded as desirable for estrus detection methods. Based on model‐predicted sensitivity, VO in both seasons, AAM in the rainy season, and AP in the dry season were acceptable. AP in the rainy season and AAM in the dry season achieved acceptable performance, whereas AP in the rainy season and AAM in the dry season performed below optimal levels. Despite these strong sensitivity values under specific conditions, overall performance varied across methods and seasons, highlighting the influence of environmental and management factors on detection efficiency (Table 3).

### 4.4. Random Effects

The random effects analysis revealed a significant cow × season interaction, with a variance of 1.83 (SD = 1.04) (see Supporting Data Table S2). This result indicates considerable individual variability in estrus response across seasons. This finding justifies including the cow × season interaction in the final model to accurately account for repeated measures under different environmental conditions. In contrast, the effect of individual cows had a zero variance estimate, suggesting that the model may have overfit the included fixed effects.

### 4.5. Performance of Estrus Detection Methods

The diagnostic performance metrics revealed an unexpected result: The specificity for the AAM system, VO, and AP was 33%, 17%, and 0%, respectively (Table 4). This low specificity indicates a failure to correctly identify cows that are not in estrus (i.e., the absence of a CL), leading to a high false‐positive rate. VO achieved the highest sensitivity (90%), followed closely by the AAM system (85%). The AP method showed comparatively lower sensitivity (74%).

**TABLE 4:** Performance of estrus detection methods (AAM, AP, and VO) compared to the physiological reference (corpus luteum) in Girolando cows (*n* = 36 confirmed observations).

Parameters	AAM	AP	VO
Sensitivity	85%	74%	90%
Specificity	33%	0%	17%
Accuracy	75%	62%	78%
Positive predictive value	85%	79%	85%
Negative predictive value	33%	0%	25%

The predictive capacity of the detection methods was evaluated using positive predictive values (PPVs), which were 85% for both AAM and VO, and 79% for AP. Overall, VO and the AAM system showed the highest accuracy (78% and 75%, respectively), while AP showed the lowest overall performance (62% accuracy) (Table 4).

## 5. Discussion

### 5.1. The Influence of Parity on Estrus Expression

Parity significantly influenced estrus detection, regardless of the season (feeding system and THI), location, or detection method. Multiparous cows exhibited a higher average probability of estrus detection (97%) compared to primiparous cows (85%) (Table 3), reflecting well‐documented differences in estrus behavior between parity groups.

Greater locomotor activity, higher movement intensity, and longer estrus duration are positively associated with improved sensor‐based detection. Tippenhauer et al. [13] reported that estrus duration is longer in multiparous than in primiparous Holstein cows. Although estrus duration was not measured in the present study, similar behavioral patterns may explain the higher detection rates observed in multiparous Girolando cows. In contrast to our results, some studies, such as Chanis [2] and Madureira et al. [14], found that primiparous cows (or those with fewer lactations) exhibit higher activity and longer estrus duration, thereby increasing sensor accuracy.

Social dominance might further explain these results. Val‐Laillet et al. [15] reported that older, multiparous dairy cows often exhibit dominant behavior, characterized by greater displacement and social interaction. This hierarchy likely influenced our results, as dominant multiparous cows may engage in more frequent movements and displacements, resulting in higher physical activity intensity. Such intensified movement patterns are more readily captured by AAM sensors, potentially explaining the superior detection rates observed in multiparous cows compared to their primiparous counterparts, who often occupy subordinate positions in the herd and may suppress their activity to avoid conflict. Ultimately, these behavioral differences affect the probability of detecting estrus with AAM systems.

### 5.2. The Challenge of Silent Estrus and Behavioral Absence

Another factor that negatively impacts AAM efficiency is the subtle expression or complete absence of physical signs of estrus, commonly known as silent ovulation. This physiological phenomenon, characterized by ovulation without detectable behavioral changes, presents a natural limit to the sensitivity of behavior‐based detection methods. Using the CL as a physiological reference (gold standard) in this study is essential because of the well‐documented disconnect between ovulation and behavioral estrus expression.

Previous studies reported that 11–17% of cows failed to show behavioral signs during postpartum ovulations [16]. This is particularly relevant in *B. indicus*‐influenced breeds, such as the Girolando, which are known for shorter, less intense, and more frequent silent heats [17]. Furthermore, Bonato et al. [17] found that estrus expression in Nellore cows was lower in primiparous than in multiparous animals, which may help explain the better detection results observed for multiparous cows in the current study.

By identifying the CL through ultrasonography 8 days after the protocol, it was possible to confirm ovulation even without standing heat or increased locomotor activity [11]. Relying on this physiological marker allows for a more accurate interpretation of performance metrics. Since AAM and VO depend solely on behavioral changes, these silent events are technically classified as false negatives. Ultimately, the occurrence of silent estrus sets a “biological ceiling” on the effectiveness of these technologies, as the lack of obvious signs underestimates the herd’s actual reproductive cyclicity.

### 5.3. Performance Variability and the Method–Season Interaction

The initial study hypothesis proposed that the AAM system could match the performance of conventional estrus detection methods (VO and AP) in grazing crossbred dairy cows. Our results not only partially supported this hypothesis but also revealed that the effectiveness of all methods, including traditional ones, varied significantly with seasonal conditions. The significant interaction between method and season confirmed this.

The AAM system clearly showed this variability: While it achieved high sensitivity during the rainy season, its performance was significantly reduced in dry conditions. This variation is likely related to differences in management and animal activity patterns.

As previously discussed, using the CL as a physiological reference was important in these situations. It helped confirm ovulation even when environmental stressors suppressed behavioral signs, preventing the overestimation of false negatives. Notably, traditional methods (VO and AP) are often criticized for their subjectivity and reliance on the observer; they also exhibited seasonal variability similar to that of the AAM system. Their consistent reliability under controlled conditions with trained observers indicates that traditional approaches can still offer reliable and accurate estrus detection when properly managed.

These findings strongly indicate that environmental conditions modulate the performance of all detection systems, regardless of their level of automation. This suggests that while precision livestock farming technologies offer advantages such as continuous monitoring and reduced labor, they may require environmental adjustments or specific calibration to achieve stable performance comparable to conventional methods.

Consequently, the AAM system can be a valid alternative, especially in situations where VO is challenging, such as nocturnal monitoring or large‐scale grazing operations. It is worth noting that precision or digital livestock farming assists the producer rather than replacing them in decision‐making [18, 19]. The integration of technology and traditional oversight is likely the most effective strategy for optimizing reproductive efficiency across variable climates.

### 5.4. Factors Affecting AAM Performance in Different Seasons

The comparative analysis highlighted key factors influencing seasonal differences in AAM sensitivity. Previous studies show that cows on pasture have more overnight locomotor activity than confined cows (1548 vs. 571 steps) [20, 21], which encourages estrus‐related behaviors such as mounting and restlessness. Our results support that AAM performance is greatly affected by feeding management; for example, the naturally higher activity levels in grazing cows increase the difference between normal behavior and estrus [10]. This seasonal variation explains the significant interaction observed in our statistical model: grazing acts as a catalyst, boosting the sensor’s signal‐to‐noise ratio. Conversely, the AAM system showed its lowest sensitivity during the dry season (73%). This decline is due to suppressed behavioral estrus signals, common in semiconfinement systems, such as limited movement in paddock resting areas and around feed bunks [22]. High animal density in these zones can greatly reduce mounting activity. Additionally, confined cows typically have shorter estrus durations and lower mounting frequencies than pasture cows, primarily due to physical discomfort and fewer opportunities for behavioral expression [23]. Additionally, heat stress may negatively affect estrus expression by decreasing dry matter intake and disrupting ovarian cyclicity [23, 24]. During the experimental period, maximum temperatures reached 35°C and 32°C in each phase, with maximum THI values of 80 and 78, respectively. These conditions may surpass the thermal comfort threshold for Girolando cows, despite their known heat tolerance. Such thermal stress can reduce the intensity and duration of estrus expression, thus contributing to the decreased efficiency of the AAM system.

### 5.5. Limitations of Traditional Methods and Implications for Management

Traditional methods such as VO, while widely used, have significant limitations, including high labor requirements, observer subjectivity, human error, and an inability to detect estrus events at night or in cows with low activity intensity [2, 25]. Similarly, AP can be triggered by nonestrus mounting, particularly in hormonally treated cows, and they are prone to issues such as device displacement or activation failure [3]. These limitations are typically linked to the frequency and duration of observation or, in the case of patches, to the absence of mounting activity and physical device loss [14, 26].

Roelofs et al. [27] noted that comparing the efficacy of VO requires detailed information on the duration, frequency, timing, and specific behaviors of the animals, as detection rates vary significantly across these variables. While VO rates can range from 38% to 89%, an observer typically detects only 50–60% of cows in estrus, a rate that is further compromised by reduced labor force and increasing herd sizes [23, 28].

The temporal distribution of estrus expression presents a major challenge for traditional methods. Sterry and Schlesser [29] showed that estrus behavior is more frequent at night, with 70% of behavioral events occurring nocturnally compared to only 30% during daylight hours. This is particularly relevant in tropical conditions. Pinheiro et al. [30] reported that in 53.8% of Nellore Zebu cows, estrus began at night (between 6:00 p.m. and 6:00 a.m.), and in 34.6% of cases, the entire estrus period occurred strictly overnight.

In the present study, VOs were conducted twice a day, between 8:00 a.m. and 4:00 p.m. Consequently, it is highly probable that cows exhibiting estrus exclusively at night remained undetected by this method. These findings underscore the logistical “blind spots” of traditional monitoring and reinforce the proposal that automated technologies are essential tools for mitigating the challenges posed by human oversight and biological variability [2].

### 5.6. The Challenge of Low Specificity and High False‐Positive Rates

The evaluated estrus detection methods showed limited ability to exclude false positives, resulting in low specificity. This performance is mainly due to the use of synchronization protocols (TAI), which significantly change herd dynamics and behavioral expression. Administering exogenous hormones, especially those that affect estradiol levels, can not only improve estrus expression but may also increase nonspecific behaviors such as social interactions and restlessness [31]. As a result, cows with a strong hormonal response tend to be more active, whereas those with a lower response may not exhibit the same behavior even under the same treatment [14]. The so‐called “herd effect” is marked by higher intensity and frequency of movement compared to natural estrus. This directly affects activity‐based monitoring, as systems might detect socially induced behaviors rather than true estrus events, leading to many false positives. For example, the AP is often triggered by increased mounting interactions among synchronized cows, regardless of whether they are in actual estrus, and VO may record secondary social activities that do not lead to ovulation. Future studies should evaluate these activity‐based monitoring systems in natural estrus conditions to better understand their effectiveness without the confounding influence of hormonal synchronization.

Furthermore, mounting behavior is not solely linked to the physiological state of estrus; it also functions as a display of social dominance or a response to overall herd excitement. In synchronized groups, the “sexually active group” (SAG) often promotes increased interaction even among nonestrual cows. AAM systems, which rely on triaxial accelerometers to detect specific motion patterns, may interpret heightened social interactions, such as restlessness, following, and mounting attempts, as estral deviations from the individual’s baseline. Since the algorithm detects the characteristic acceleration of these movements rather than a direct physiological marker, socially induced hyperactivity can be easily mistaken for true proestrus or estrus behavior, thus reducing specificity.

### 5.7. Practical Applications and Management Guidelines

Previous studies of physical activity monitoring systems in pasture‐based dairy cattle have reported sensitivities ranging from 72% to 90% and PPVs from 67% to 78% [32]. For effective practical application, AAM systems must achieve both high sensitivity to eliminate false negatives and high specificity to confirm true estrus events, thereby preventing unnecessary breeding costs [33]. Low specificity is particularly detrimental to profitability, as it increases the number of inseminations per conception. The PPV is highly relevant to producers. A PPV of 85% indicates that if the AAM system issues an alert, there is an 85% chance the cow is genuinely in estrus.

During the dry season, the AAM system in this study showed reduced sensitivity (73%), which is a significant disadvantage for operations using high‐value or sexed semen. Conversely, the AP method demonstrated high sensitivity (95%) during the same period. This discrepancy underscores the need for combined detection protocols, in which AP results are supplemented with methods such as periodic ultrasonography to confirm estrus before insemination.

To maximize reproductive efficiency under variable seasonal conditions, we propose the following guidelines for the dry season (semiconfinement): (1) Adopt combined detection protocols (sensors paired with increased daytime VO). (2) Prioritize multiparameter sensors (tracking activity, rumination, and vaginal temperature) to compensate for lower locomotor intensity. For the rainy season (grazing). (3) Focus on “filtering” alerts. Integrate physiological indicators (progesterone concentration) and correlate sensor data with typical behavioral patterns to reduce the impact of false positives due to social displacements. (4) Clinical support: Conduct weekly ultrasound examinations for cows with DIM > 45 to identify noncycling animals or confirm estrus in cases of ambiguous sensor alerts.

### 5.8. Future Directions

Based on our findings and the evaluated experimental conditions, the AAM system appears most effective in rainy season grazing operations, where its high sensitivity ensures that few estrus events are missed. However, its use under these conditions requires refined algorithms to mitigate the false‐alarm rate associated with increased social displacement. Future research should (1) involve larger cohorts across diverse management systems; (2) refine AAM configuration settings, specifically estrus intensity and duration thresholds, to improve PPV and reduce false positives; and (3) conduct a comprehensive economic analysis of the impact of false positives versus missed estruses.

A critical consideration for future studies is the influence of hormonal synchronization. Therefore, future research must validate these results in animals exhibiting natural estrus to determine whether the observed behavioral responses are truly representative of physiological estrus or are confounded by synchronized social interactions.

## 6. Conclusion

Parity and the method × season interaction significantly influenced estrus detection. While the VO and AAM systems showed high sensitivity, their low specificity remains a key limitation, especially when synchronization protocols are used, which can lead to increased false positives. This study provides a foundation for future system improvements and adoption in extensive dairy systems, aiming to enhance reproductive efficiency and herd productivity. For optimal use in variable grazing systems, the AAM system needs refined algorithms to differentiate true estrus from nonestrus social behaviors.

## Author Contributions

Sara Adna Santos De Oliveira wrote the manuscript, participated in the field data collection, and organized the database. Ricardo Alamino Figueiredo contributed to the critical review of the manuscript. João Henrique Moreira Viana contributed to the critical review of the manuscript. Livia Loiola Dos Santos Féres translated the article and assisted in the revision. Luiz Fernando Rodrigues Féres assisted in the manuscript revision. Ricarda Maria Dos Santos contributed to the critical review of the manuscript. Carlos Frederico Martins contributed to the critical review of the manuscript and implemented experimental protocols and management practices with the animals. Isabel Cristina Ferreira coordinated the entire experimental line, supervised the methodology and field data analysis, conducted field data collection, performed statistical analyses, and assisted in the processing of field data.

## Funding

This work was supported by the Fundação de Apoio à Pesquisa do Distrito Federal (1556/2017) and Coordenação de Aperfeiçoamento de Pessoal de Nível Superior.

## Conflicts of Interest

The authors declare no conflicts of interest.

## Supporting Information

Additional supporting information can be found online in the Supporting Information section.

## Supporting information


**Supporting Information** Table S1. *p* values for the main fixed effects (method, location, season, and parity) and for the interactions tested individually in each model. The individualized testing was performed because the full model, including all main effects and interactions, presented convergence limitations. Table S2. The structure used to model individual variability across seasons in a repeated‐measures design with the same animals.

## Data Availability

The data that support the findings of this study are available from the corresponding author upon reasonable request.
