# Supplementary material for: Parity and Season Affect the Performance of an Automated Activity Monitoring System for Estrus Detection in Grazing Girolando Cows
Source: Vet Med Int. 2026 May 29;2026:8201912. doi: 10.1155/vmi/8201912 (PMC13239506; doi:10.1155/vmi/8201912)
Supplement: Supplementary file 1 — Supporting Information Table S1. p values for the main fixed effects (method, location, season, and parity) and for the interactions tested individually in each model. The individualized testing was performed because the full model, including all main effects and interactions, presented convergence limitations. Table S2. The structure used to model individual variability across seasons in a repeated‐measures design with the same animals. [file VMI-2026-8201912-s001.docx]

Suplementary dates

Table S1-P-values for the main fixed effects (method, location, season, and parity) and their respective interactions

|  | P-value |
| --- | --- |
| Method | 0.8809 |
| Location | 0.2286 |
| Season | 0.9011 |
| Parity | 0.0332 |
| Method*location | 0.6220 |
| Method*season | 0.0012 |
| Method *parity | 0.9345 |
| Location*parity | 0.6539 |
| Location*season | 0.1419 |
| Season*parity | 0.9022 |

Table S2 -Covariance parameter estimates and standard errors for cow and cow × season interaction effects

| Cov Parm | Subject | Estimate | Standard |
| --- | --- | --- | --- |
| Intercept | Cow*season | 1.8254 | 1.0446 |
| Intercept | cow | 0 | . |
